# Cryptic Species? Patterns of Maternal and Paternal Gene Flow in Eight Neotropical Bats

**DOI:** 10.1371/journal.pone.0021460

**Published:** 2011-07-26

**Authors:** Elizabeth L. Clare

**Affiliations:** Biodiversity Institute of Ontario, Department of Integrative Biology, University of Guelph, Guelph, Ontario, Canada; University of Florence, Italy

## Abstract

Levels of sequence divergence at mitochondrial loci are frequently used in phylogeographic analysis and species delimitation though single marker systems cannot assess bi-parental gene flow. In this investigation I compare the phylogeographic patterns revealed through the maternally inherited mitochondrial COI region and the paternally inherited 7^th^ intron region of the *Dby* gene on the Y-chromosome in eight common Neotropical bat species. These species are diverse and include members of two families from the feeding guilds of sanguivores, nectarivores, frugivores, carnivores and insectivores. In each case, the currently recognized taxon is comprised of distinct, substantially divergent intraspecific mitochondrial lineages suggesting cryptic species complexes. In *Chrotopterus auritus*, and *Saccopteryx bilineata* I observed congruent patterns of divergence in both genetic regions suggesting a cessation of gene flow between intraspecific groups. This evidence supports the existence of cryptic species complexes which meet the criteria of the genetic species concept. In *Glossophaga soricina* two intraspecific groups with largely sympatric South American ranges show evidence for incomplete lineage sorting or frequent hybridization while a third group with a Central American distribution appears to diverge congruently at both loci suggesting speciation. Within *Desmodus rotundus* and *Trachops cirrhosus* the paternally inherited region was monomorphic and thus does not support or refute the potential for cryptic speciation. In *Uroderma bilobatum*, *Micronycteris megalotis* and *Platyrrhinus helleri* the gene regions show conflicting patterns of divergence and I cannot exclude ongoing gene flow between intraspecific groups. This analysis provides a comprehensive comparison across taxa and employs both maternally and paternally inherited gene regions to validate patterns of gene flow. I present evidence for previously unrecognized species meeting the criteria of the genetic species concept but demonstrate that estimates of mitochondrial diversity alone do not accurately represent gene flow in these species and that contact/hybrid zones must be explored to evaluate reproductive isolation.

## Introduction

The Neotropical regions of Central and South America contain extremely high bat species richness [1] and have received extensive taxonomic scrutiny [2]–[6]. The taxonomic diversity of bats in the New World increases with decreasing latitude except for vespertilionids, which are more species rich at mid-latitudes [1]. The rate of increase in diversity varies; with species richness of phyllostomids increasing dramatically towards the equator while the diversity of molossids increases more slowly [1]. Through continuous taxonomic investigation, new bat species are described from these regions commonly [7], [8] and extensive cryptic diversity is suspected [6], [8]–[12].

Recent technological advances have led to the rapid accumulation of genetic data from homologous gene regions in many animal species. DNA barcoding [13] relies on sequence diversity at the 5′ end of the mitochondrial cytochrome *c* oxidase subunit 1 gene (COI) to provide a method of species identification and to advance species discovery by examining patterns of inter- and intraspecific diversity at this locus. While this method is not a species concept, it is an efficient hypothesis generator and has led to numerous proposed cryptic species. Previous analyses [10], [11] have demonstrated the utility of barcoding for species recognition in bats and have identified several Neotropical bat taxa showing substantial intraspecific mitochondrial divergences (2–14%) within small geographic regions and larger continental surveys incorporating mitochondrial DNA [6], [12] have revealed additional examples. Two alternative hypotheses may explain these patterns. First, deep mitochondrial splits may represent phylogeographic structuring and potentially the effects of female philopatry. Alternatively, these splits may reflect unrecognized species. While both phylogeographic structuring and unrecognized speciation are plausible, neither can be excluded using maternally inherited mitochondrial DNA alone.

Habitat-restriction among Neotropical species may have led to genetic structuring and rapid speciation during the Pleistocene when climatic fluctuations caused continual shifts in forest ranges and recurrent cycles of expansion and contraction of populations in a complex system of refugia [14]. However, bat species diversity does not appear to be closely related to habitat and their higher vagility likely leads to a release from habitat restriction [1]. Substantial population structure in bats has been noted among island populations while those with continental distributions show variable patterns of phylogeographic differentiation [15]. In many phyla, particularly invertebrates, geographic structure is inversely correlated with dispersal ability [16], [17] although no similar pattern has been found between wing aspect ratio (as a predictor of dispersal ability) and mitochondrial molecular diversity in Neotropical bats (E. Clare, unpublished data). Phylogeographic structuring between Central and South America is more consistent among bat taxa though the lack of fossil evidence combined with high dispersal capability has limited our ability to explain observed phylogeographic structure [17]. Zoogeographical analyses suggest that bats from west of the Andes are frequently more similar to Central American fauna than they are to other South American populations east of the Andes [17], [18] and a similar pattern has been noted in birds [19] suggesting that many dispersals events have occurred along the Andes cordilleras. In particular, comparable phylogeographic patterns have been observed in the phyllostomids *Desmodus rotundus* and species of *Carollia* indicating the importance of mountains as a dispersal barrier even in highly mobile taxa [17].

Most mammal species appear to meet the conditions for recognition under the Biological Species Concept (BSC) [20]. Interspecific hybridization is uncommon and only three cases have been reported among described bat species, involving *Myotis myotis* and *M. blythii* [21], *Pteropus alecto* and *P. poliocephalus* [22] and historically between populations of *Rhinolophus yunnanensis* and *R. pearsoni* [23]. A Genetic Species Concept (GSC) has been proposed [6] for mammals based on the Bateson-Dobzhansky-Muller model [24]. The GSC places emphasis on species recognition by genetic divergence rather than reproductive isolation, suggesting genetic changes accumulate in lineages until the integrity of the gene pools as separate entities is established. In practice, Baker and Bradley's GSC [6] is similar to a relaxed BSC [25] which allows for small amounts of gene flow between substantially reproductively isolated groups although the emphasis suggests gene flow is rare in the BSC but expected in the GSC. It is unclear whether this predicted gene flow is simply undetected between many species identified by traditional morphological methods, or will exist between species identified by the GSC because they are delimited earlier in the speciation process and are thus less reproductively isolated. The GSC has the advantage of being applicable to allopatric populations where tests of reproductive isolation are not practical. Using the same theoretical framework, Bradley and Baker [9] set out a series of criteria for evaluating the taxonomic implications of mitochondrial sequence divergence: values <2% were indicative of intraspecific variation, values between 2 and 11% were indicative of conspecific variation (requiring additional taxonomic scrutiny when found intraspecifically) while intraspecific values >11% likely indicate the presence of undescribed species. While evaluating mtDNA divergence can be an extremely useful hypothesis generator, it is also clear that sequence divergence does not necessarily equate with gene flow, though few studies have evaluated these criteria. This may be particularly problematic in mammals where female philopatry and male mediated gene flow are common and a strict reliance on mtDNA can be problematic. It is not clear whether this pattern can persist for thousands (or millions) of years generating genetic structure thus evaluation of mtDNA patterns is essential.

The status of potential cryptic species first revealed as deeply divergent intraspecific mitochondrial lineages can be assessed using alternate lines of evidence such as ecological data [26], morphology [7], or additional gene regions, particularly with different modes of inheritance [27]. Here I examine phylogeographic patterns in COI and test for gene flow between intraspecific lineages in eight widespread Neotropical bat species using a second, paternally inherited, gene region which includes a 3′ portion of the 7^th^ exon, the 7^th^ intron and a 5′ portion of the 8^th^ exon of the Y-chromosome *Dby* gene (also known as *Ddx3y*, DEAD box RNA helicase Y) [28]. Y-chromosome regions have obvious advantages for comparisons to mtDNA as they are fast evolving, non-recombining and provide an exclusively paternal measure of gene flow while standard nuclear regions tend to evolve more slowly and recombination makes separating historical parental contributions difficult.

In a separate analysis [12] 44 of 163 examined Neotropical bat species were identified as containing distinct mitochondrial lineages. Here I attempt to differentiate cases where deeply divergent mitochondrial lineages may reflect phylogeographic structure from those which may represent cryptic species by comparing the patterns of gene flow suggested by mtDNA and the Y-chromosome region in eight cases. These species are widely distributed, taxonomically diverse and include members of two families with varied ecological roles including sanguivores, nectarivores, frugivores, carnivores and insectivores. I test the hypothesis that divergent intraspecific mitochondrial lineages in these eight species represent segregated gene pools with independent evolutionary histories: if so 1) these two independently evolving gene regions (mitochondrial genome and Y-chromosome) should show congruent patterns of genetic divergence and 2) the genetic associations should be maintained with both allopatric and sympatric contemporary distributions. In addition, I evaluate the results against the criteria set out by Bradley and Baker [9] to see how often sequence divergence was predictive of gene flow patterns.

## Methods

### Sequence Acquisition and Analysis

I generated COI sequences for eight bat species widely distributed in continental Central and South America (Table 1) following the methods of Clare et al. [10], [12] and Borisenko et al. [11]. For males, I generated sequences from the *Dby* 7^th^ intron region of the Y-chromosome following the same protocols with the primers described in Lim et al. [28] and Lim [29]. All sequences were derived from vouchered specimens held at the Royal Ontario Museum (ROM), Toronto, Canada. I edited COI sequences using SeqScape v.2.1.1 (Applied Biosystems) and sequences from the *Dby* 7^th^ intron region in Sequencher v.4.5 (Gene Codes). I manually aligned all sequences in BioEdit v.7.0.9 (Ibis BioSciences). Sequences and specimen collaterals (sampling location, GPS co-ordinates of collection, voucher number etc.) are available within the “Bats of the Neotropics” project in the Barcode of Life Data Systems (BOLD, www.barcodinglife.org). GenBank accessions and associated BOLD and ROM numbers are found in Table S1.

**Table 1:** Distribution of samples acquired for species of bats used in this study. N = total sample size.

	Species	Ecuador	Brazil	Guyana	Suriname	El Salvador	Guatemala	Panama	Venezuela	Mexico	Costa Rica	N
1	*Chrotopterus auritus*	2	3	52	3		1			3		64
2	*Saccopteryx bilineata*	12		75	43	1	2					133
3	*Glossophaga soricina*	3	1	128	13		20	2	3	17		187
4	*Desmodus rotundus*	11	8	64	3	2	2	2	2	6	3	103
5	*Trachops cirrhosus*	23		86	8		2	1	6	1		127
6	*Uroderma bilobatum*	37		77	9			7			4	134
7	*Micronycteris megalotis*	7		27	2			2	2	4		44
8	*Platyrrhinus helleri*	33		76	21		2	4			4	140

For comparison with the criteria of Baker and Bradley [9] I calculated the mean and range of intraspecific sequence variation using the Kimura two-parameter (K2P) model of base substitution [30] and the mean K2P sequence diversity between intraspecific mitochondrial groups using MEGA v.4.0 [31].

### Phylogenetic Reconstructions

For each COI dataset I constructed a 95% confidence limit haplotype network using statistical parsimony in TCS v.1.13 [32]. In all analyses, “groups” are defined as independent (unconnected) networks at the 95% confidence interval with the exception of *Glossophaga soricina* where groups are defined by phylogenetic analysis for reasons discussed below. For COI data I computed hierarchical likelihood ratio tests (hLRTs) for 56 models of sequence evolution in MODELTEST [33] executed in PAUP v. 4b10 [34] and interpreted the results with MtGui to select the most appropriate model of sequence evolution for each set of COI sequences for each bat species. I constructed maximum likelihood phylogenies (ML) using PhyML 3.0 [35] as implemented by the ATGC Montpellier Bioinformatics Platform (http://www.atgc-montpellier.fr/phyml/) using the most appropriate model of sequence evolution. Branch support was calculated using the non-parametric Shimodaira-Hasegawa-like (SH-like) approximate likelihood ratio test (aLRT). For each COI sequence set I also constructed a Bayesian phylogeny in MrBayes 3.1.2 [36] using the most appropriate model of sequence evolution. Analyses were performed for 1,000,000 generations for every 10 specimens in the analysis. Runs were sampled every 50 generations with a burn-in of 2000 generations. All displayed COI trees are ML topologies with SH-like branch supports with the exception of *D. rotundus* and *G. soricina* where both ML and Bayesian phylogenies are displayed. For each *Dby* 7^th^ intron region dataset, I examined sequences in BioEdit v.7.0.9 (Ibis BioSciences) to identify fixed characters between intraspecific groups identified by COI regions. I visualize these groups using UPGMA trees constructed in MEGA v.4.0 [37].

## Results

The mean and range of K2P sequence divergence per species (Table 2) indicates that in all cases, except *U. bilobatum*, mean sequence divergence falls in the range of Bradley and Baker's [9] second criteria - species where intraspecific variation suggests cryptic speciation in need of greater taxonomic scrutiny. Maximum sequence divergences were more variable with the smallest in *U. bilobatum* (4.2%) and the largest in *C. auritus* (16%). The estimated number of intraspecific mitochondrial lineages ranged from 3–9 and the range of mean pairwise K2P sequence divergences between groups varied from 1.1% in *U. bilobatum* to 15.2% in *C. auritus* (Table 2).

**Table 2:** Sequence divergence (K2P) at the COI region within and between intraspecific groups identified in this study.

	Species	Mean (range) % sequence divergence for all sequences	Number of lineages	Range of mean pairwise sequence divergences between identified groups
1	*Chrotopterus auritus*	3.5 (0–16.0)	3	10.1–15.2
2	*Saccopteryx bilineata*	2.5 (0–9.5)	3	7.4–8.6
3	*Glossophaga soricina*	2.7 (0–5.9)	3	2.6–4.8
4	*Desmodus rotundus*	3.5 (0–6.6)	6	4.2–6.1
5	*Trachops cirrhosus*	3.9 (0–8.4)	9	2.3–8.4
6	*Uroderma bilobatum*	1.1 (0–4.2)	2	1.3–3.1
7	*Micronycteris megalotis*	4.2 (0–7.7)	9	1.1–6.9
8	*Platyrrhinus helleri*	2.4 (0–5.8)	4	3.1–5.1

An HKY model of sequence evolution [38] best fit COI sequences in all cases except *Platyrrhinus helleri* where a TrN model [39] was most appropriate (hLRT and AIC suggested similar evolutionary models in most cases, though hLRT tended to be more conservative and was employed here). In all cases, an analysis using a 95% confidence haplotype network revealed numerous unconnected networks within currently recognized species. Reticulations are indicated with dashed lines demonstrating alternative hypothetical connections.

**Figure 1:**
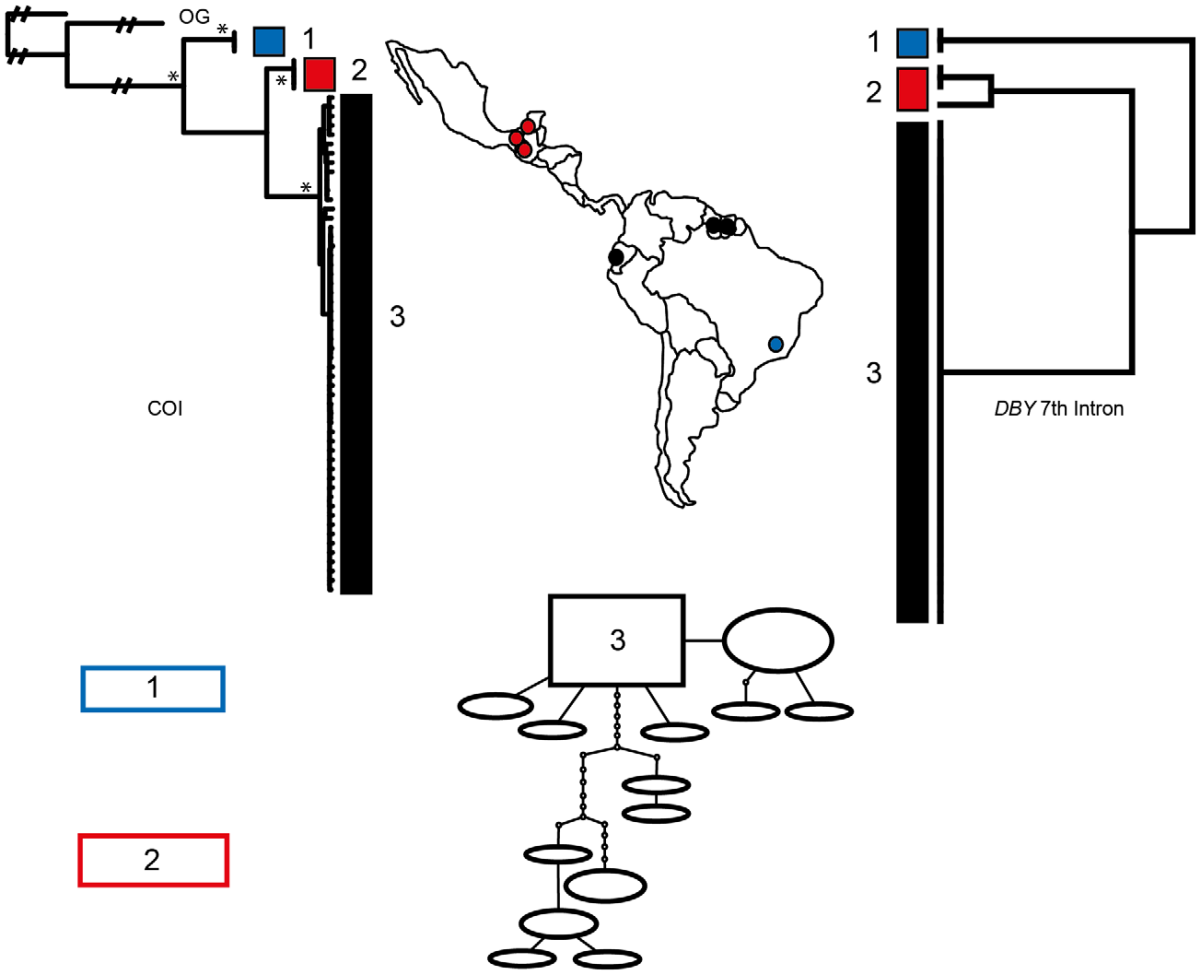
Haplotype maps and phylogenetic reconstruction of COI and the 7^th^ intron of the *Dby* gene in *Chrotopterus auritus* with sampling distributions. In the statistical parsimony networks each circle represents a single haplotype with circle size scaled by haplotype frequency. Squares indicate the most common haplotype in the network. The COI tree represents the maximum likelihood phylogeny of mitochondrial lineages. Branch supports represent non-parametric Shimodaira-Hasegawa-like (SH-like) values, those equal or greater than 95% are indicated with an asterisk. Y-chromosome intron data is depicted using a UPGMA diagram. Colour coding of branches and haplotype networks matches the sampling distribution.

**Figure 2:**
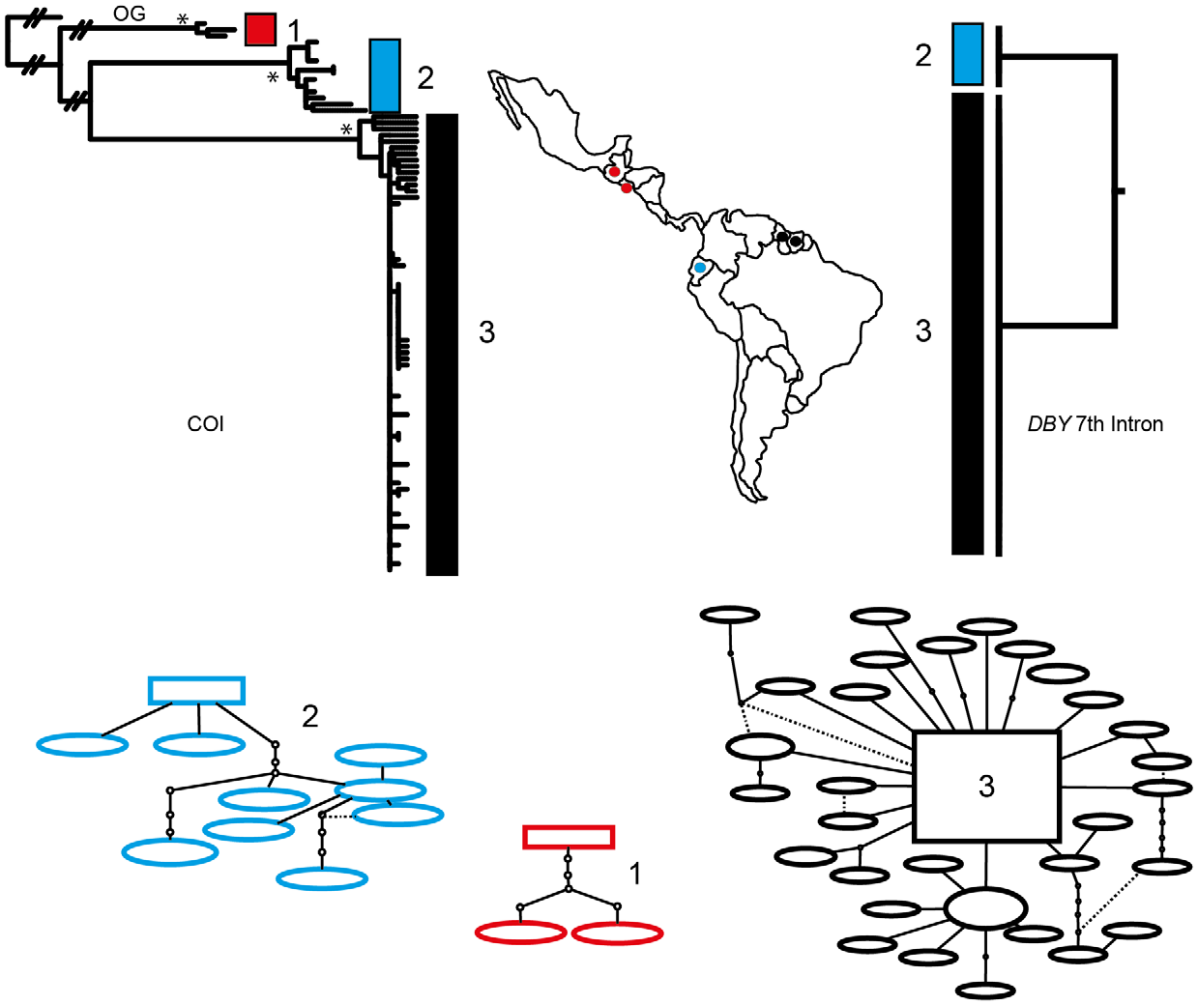
Haplotype maps and phylogenetic reconstruction of COI and the 7^th^ intron of the *Dby* gene in *Saccopteryx bilineata* with sampling distributions. In the statistical parsimony networks each circle represents a single haplotype with circle size scaled by haplotype frequency. Squares indicate the most common haplotype in the network. The COI tree represents the maximum likelihood phylogeny of mitochondrial lineages. Branch supports represent non-parametric Shimodaira-Hasegawa-like (SH-like) values, those equal or greater than 95% are indicated with an asterisk. Y-chromosome intron data is depicted using a UPGMA diagram. Colour coding of branches and haplotype networks matches the sampling distribution.

**Figure 3:**
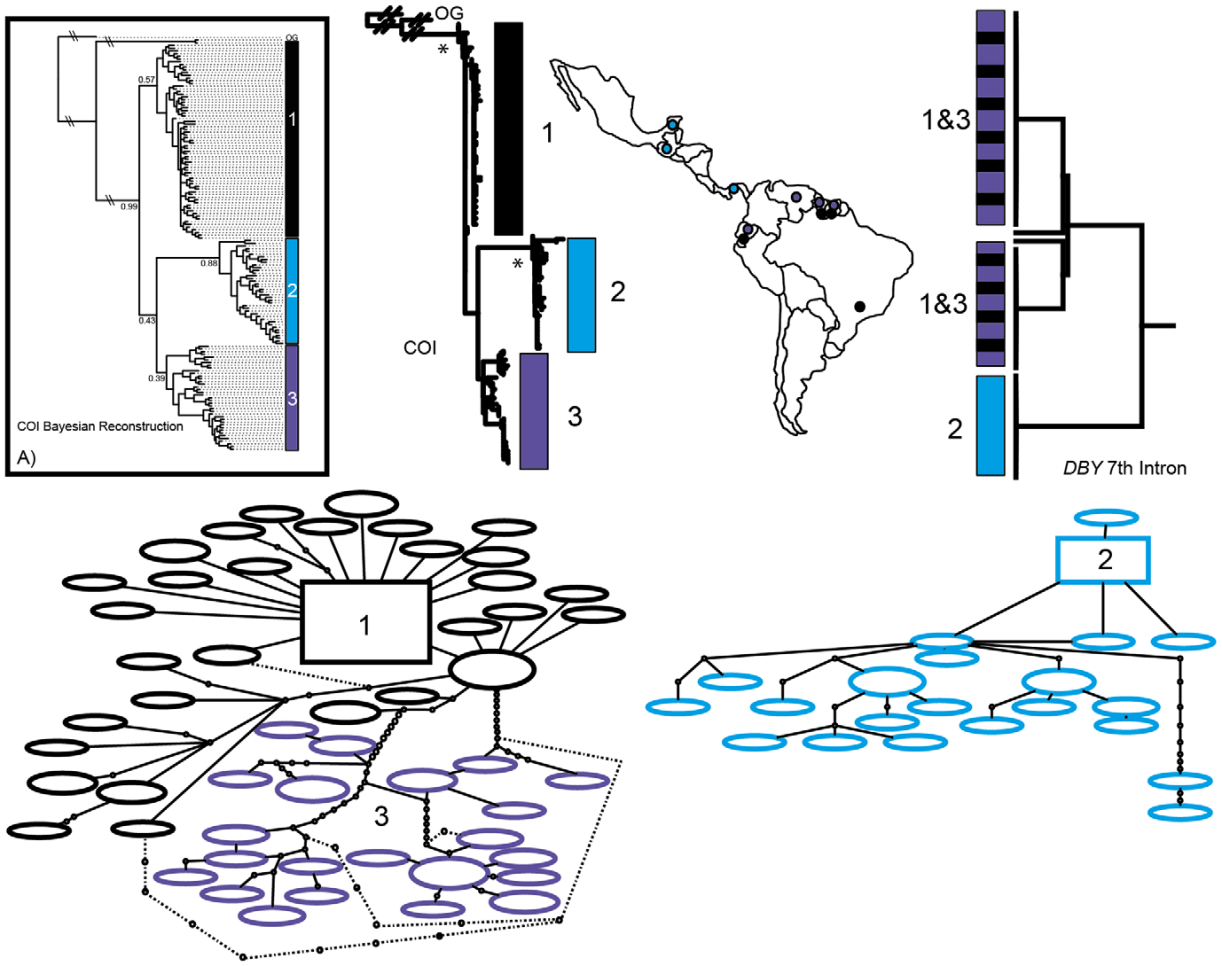
Haplotype maps and phylogenetic reconstruction of COI and the 7^th^ intron of the *Dby* gene in *Glossophaga soricina* with sampling distributions. In the statistical parsimony networks each circle represents a single haplotype with circle size scaled by haplotype frequency. Squares indicate the most common haplotype in the network. The COI tree represents the maximum likelihood phylogeny of mitochondrial lineages. Branch supports represent non-parametric Shimodaira-Hasegawa-like (SH-like) values, those equal or greater than 95% are indicated with an asterisk. Inset A) is the Bayesian reconstruction. Y-chromosome intron data is depicted using a UPGMA diagram. Colour coding of branches and haplotype networks matches the sampling distribution.

Phylogenetic reconstruction of COI sequences from *Chrotopterus auritus* (Figure 1), *Saccopteryx bilineata* (Figure 2) and *Glossophaga soricina* (Figure 3) each contain three distinct mitochondrial lineages. In *C. auritus* and *S. bilineata* each lineage corresponds to a distinct network (Figure 1 and 2). In *G. soricina* (Figure 3) haplotype networks recover two distinct groupings which are restricted to Central and South America respectively. The Central American network corresponds to lineage 2 in the *G. soricina* phylogenies while the South American network contains both lineage 1 and 3 making this network appear paraphyletic within the phylogenetic reconstructions. Group 1 was difficult to resolve as reciprocally monophyletic in the ML phylogeny but was recovered in the Bayesian phylogeny (Figure 3). Specimens in this group show considerable sequence divergence from lineage 2 and 3 (greater than 2.5%) and may be distinct within the haplotype network depending on how reticulations are resolved. I thus considered three groups of *G. soricina* based on the Bayesian phylogeny rather than the two identified by discrete networks.

**Figure 4:**
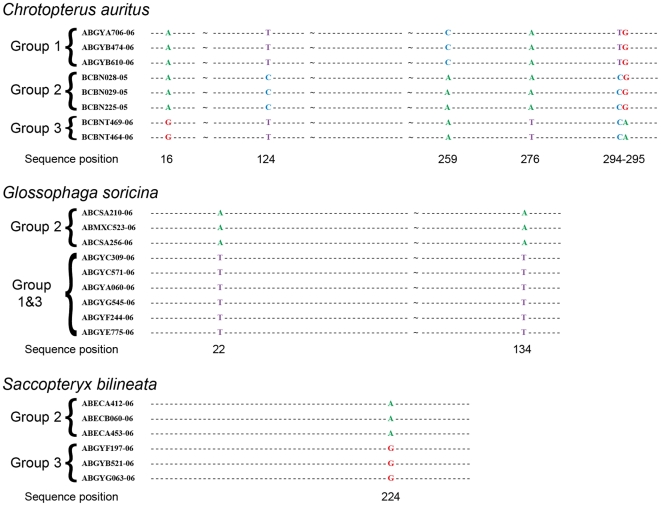
Characters in the *Dby* 7^th^ intron which differentiate mitochondrial lineages in three species of Neotropical bats. Base pair references are given below the sequences. ∼indicates removed sequence positions that contain no polymorphisms. Sequences are not aligned between species and thus base pair numbers do not correspond. In *C. auritus* group 1 n = 3, group 2 n = 2, group 3 n = 28. In *S. bilineata* group 2 n = 4, group 3 n = 35. In *G. soricina* group 1 n = 29, group 2 n = 15, group 3 n = 18.

In all three of these taxa, distinct mitochondrial groups are supported by fixed substitutions in the *Dby* 7^th^ intron (Figure 4) with the exception of group 1 and 3 in *G. soricina* which cannot be discriminated at this locus. I found evidence of shared Y-chromosome haplotypes between *G. soricina* groups 1 and 3 which appear to occupy similar geographic distributions (Figure 3). In *S. bilineata*, group 1 contained only females so is not evaluated here. A representative set of sequences from each group is displayed (Figure 4). The total number of males sequenced for each group is indicated.

**Figure 5:**
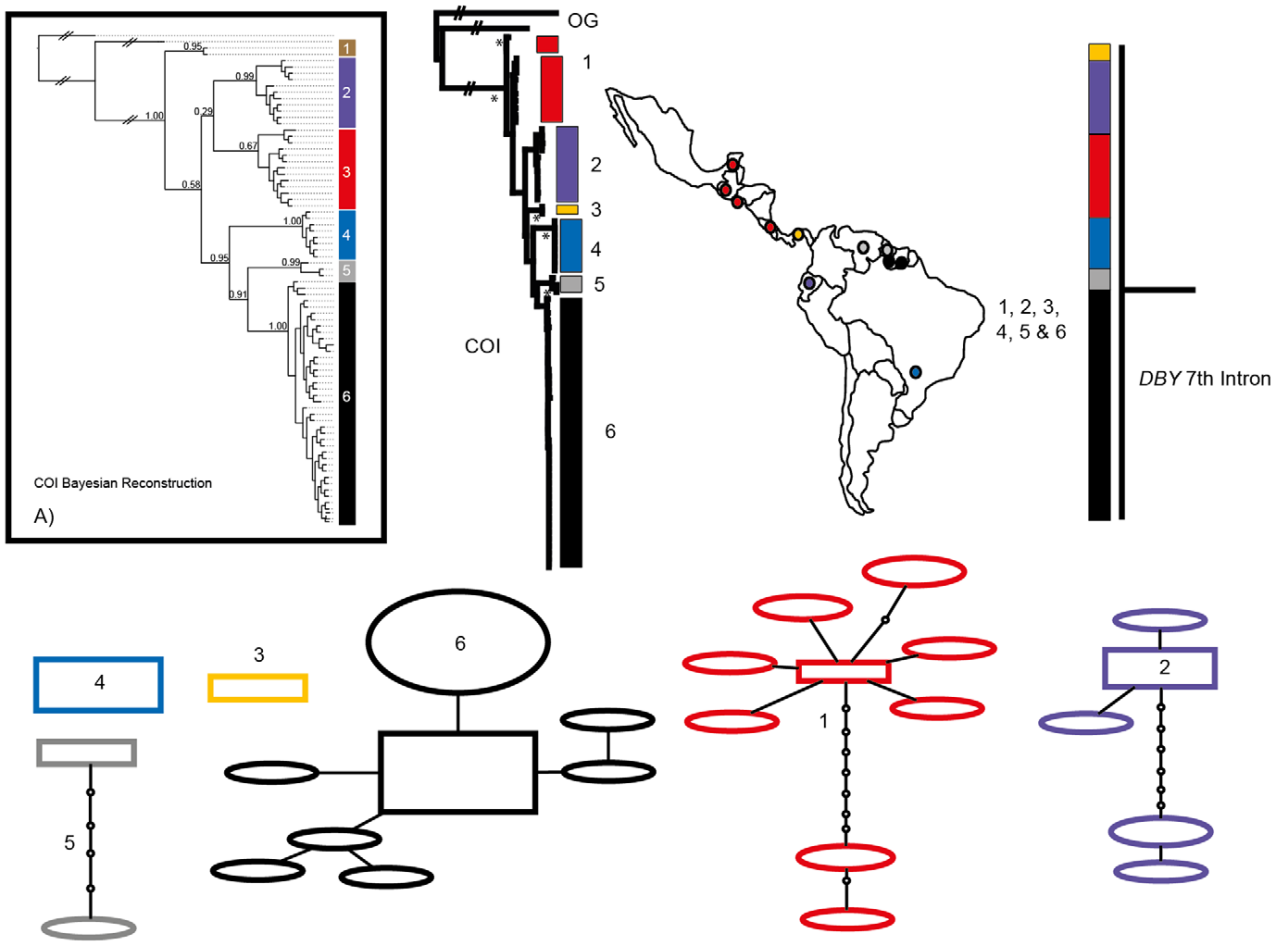
Haplotype maps and phylogenetic reconstruction of COI and the 7^th^ intron of the *Dby* gene in *Desmodus rotundus* with sampling distributions. In the statistical parsimony networks each circle represents a single haplotype with circle size scaled by haplotype frequency. Squares indicate the most common haplotype in the network. The COI tree represents the maximum likelihood phylogeny of mitochondrial lineages. Branch supports represent non-parametric Shimodaira-Hasegawa-like (SH-like) values, those equal or greater than 95% are indicated with an asterisk. Inset A) is the Bayesian reconstruction. Y-chromosome intron data is depicted using a UPGMA diagram. Colour coding of branches and haplotype networks matches the sampling distribution.

**Figure 6:**
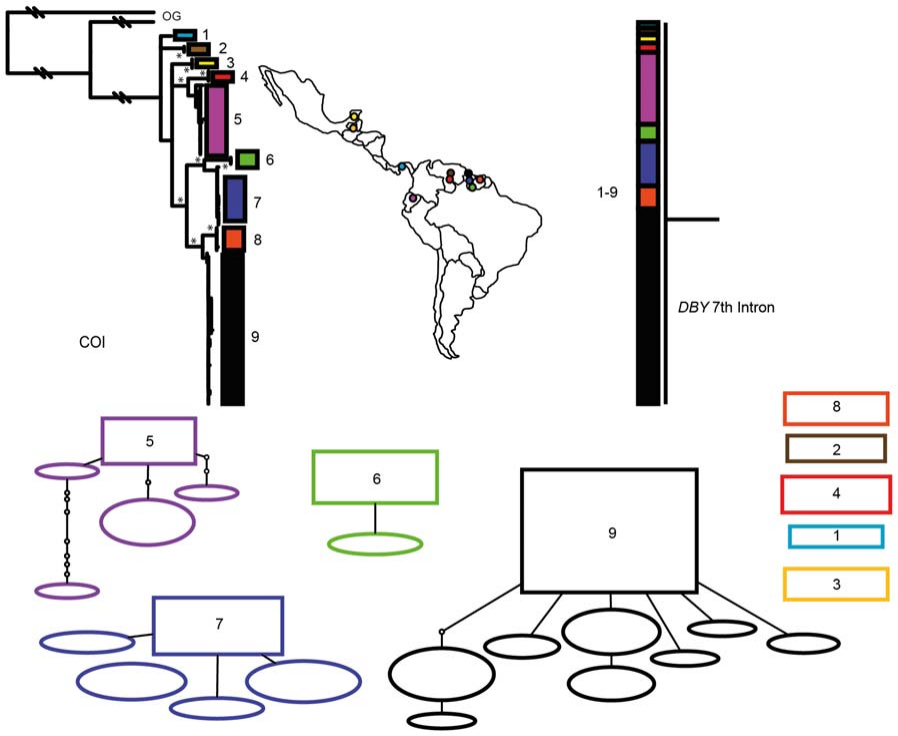
Haplotype maps and phylogenetic reconstruction of COI and the 7^th^ intron of the *Dby* gene in *Trachops cirrhosus* with sampling distributions. In the statistical parsimony networks each circle represents a single haplotype with circle size scaled by haplotype frequency. Squares indicate the most common haplotype in the network. The COI tree represents the maximum likelihood phylogeny of mitochondrial lineages. Branch supports represent non-parametric Shimodaira-Hasegawa-like (SH-like) values, those equal or greater than 95% are indicated with an asterisk. Y-chromosome intron data is depicted using a UPGMA diagram. Colour coding of branches and haplotype networks matches the sampling distribution.

In *Desmodus rotundus* (Figure 5) and *Trachops cirrhosus* (Figure 6) six and nine networks were identified respectively corresponding to distinct mitochondrial lineages however, in both cases, the *Dby* 7^th^ intron region was monomorphic. Group 1 in *D. rotundus* is not recovered as a reciprocally monophyletic lineage in the ML reconstruction though it is in the Bayesian phylogeny (Figure 5).

**Figure 7:**
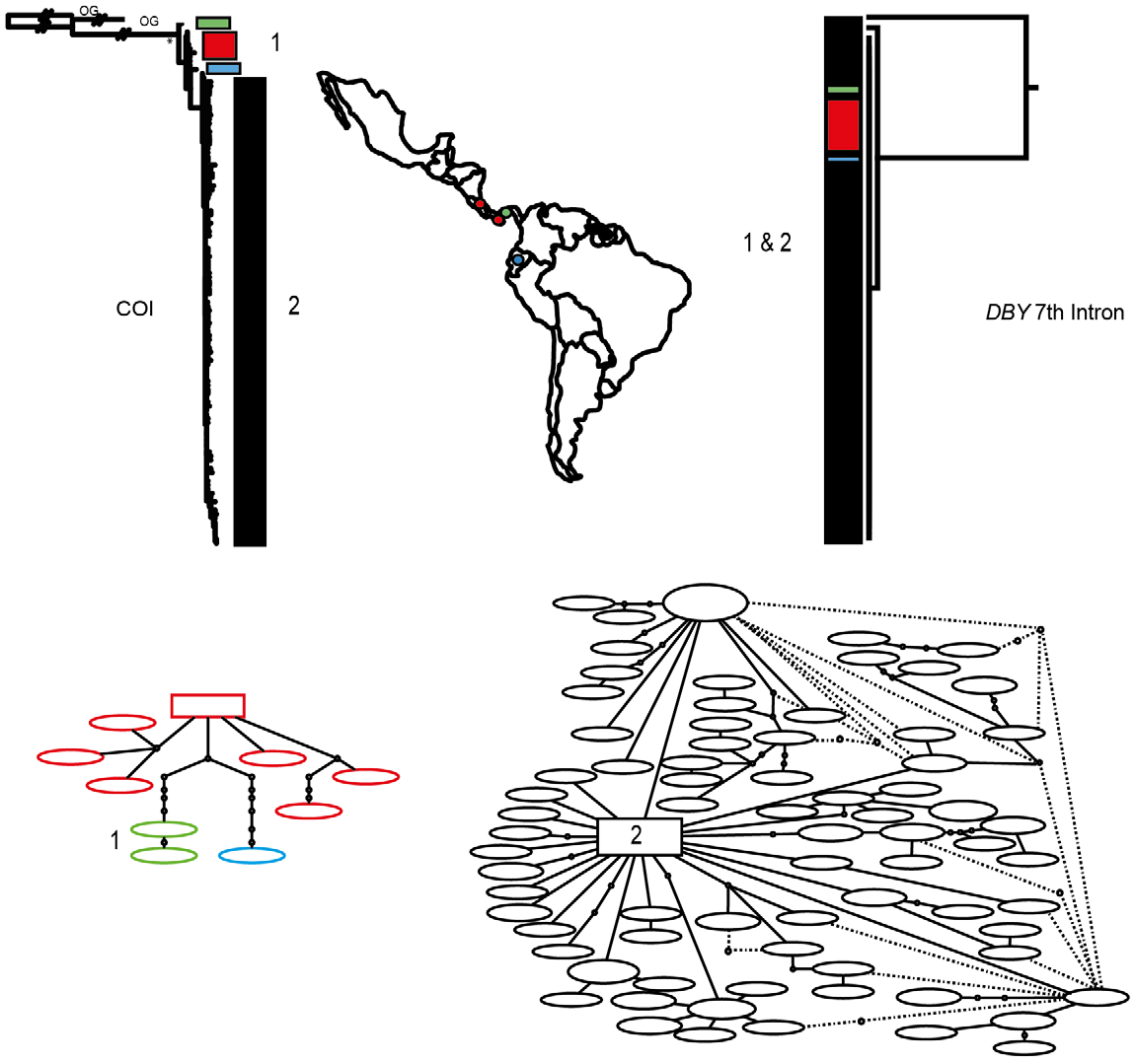
Haplotype maps and phylogenetic reconstruction of COI and the 7^th^ intron of the *Dby* gene in *Uroderma bilobatum* with sampling distributions. In the statistical parsimony networks each circle represents a single haplotype with circle size scaled by haplotype frequency. Squares indicate the most common haplotype in the network. The COI tree represents the maximum likelihood phylogeny of mitochondrial lineages. Branch supports represent non-parametric Shimodaira-Hasegawa-like (SH-like) values, those equal or greater than 95% are indicated with an asterisk. Y-chromosome intron data is depicted using a UPGMA diagram. Colour coding of branches and haplotype networks matches the sampling distribution.

**Figure 8:**
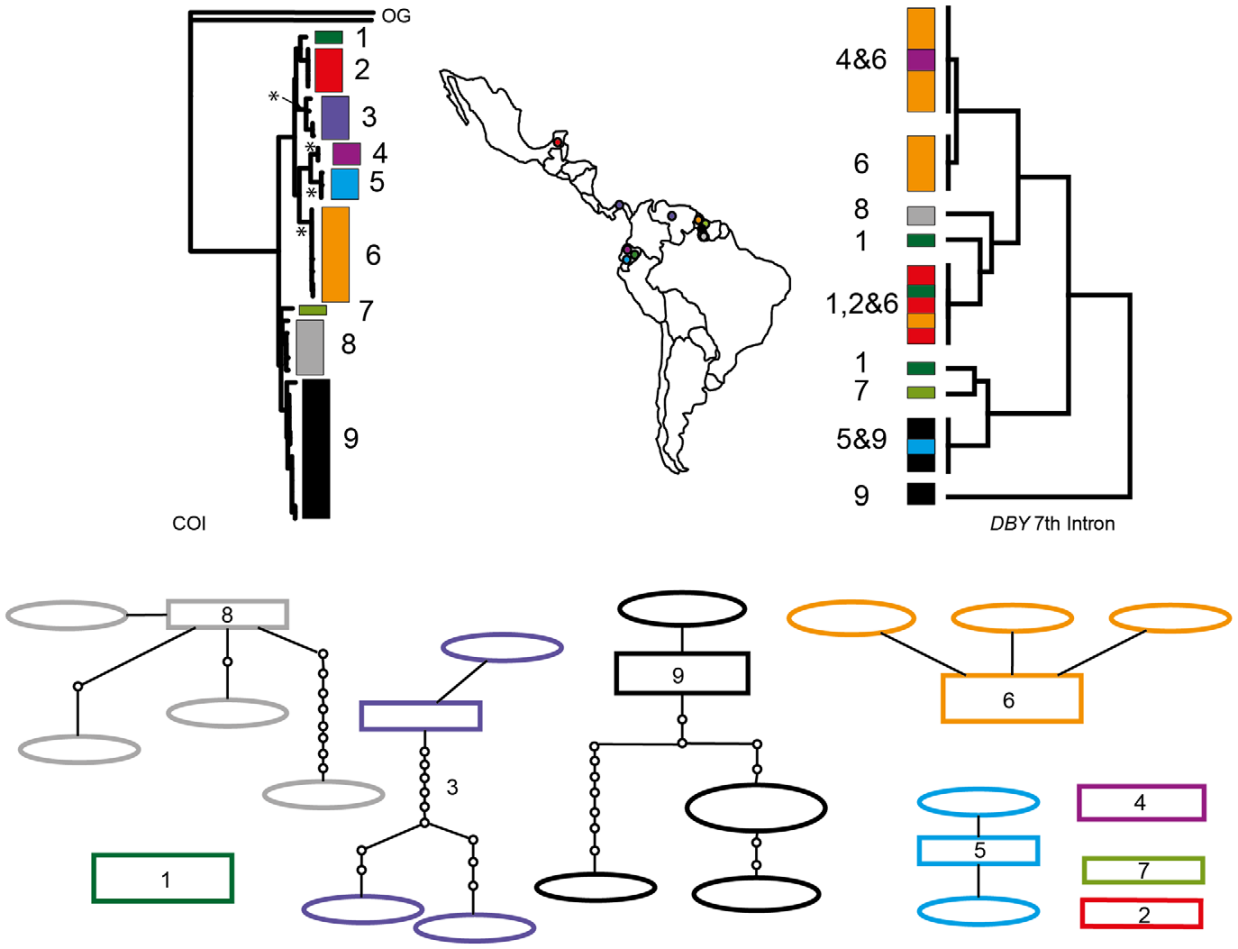
Haplotype maps and phylogenetic reconstruction of COI and the 7^th^ intron of the *Dby* gene in *Micronycteris megalotis* with sampling distributions. In the statistical parsimony networks each circle represents a single haplotype with circle size scaled by haplotype frequency. Squares indicate the most common haplotype in the network. The COI tree represents the maximum likelihood phylogeny of mitochondrial lineages. Branch supports represent non-parametric Shimodaira-Hasegawa-like (SH-like) values, those equal or greater than 95% are indicated with an asterisk. Y-chromosome intron data is depicted using a UPGMA diagram. Colour coding of branches and haplotype networks matches the sampling distribution.

**Figure 9:**
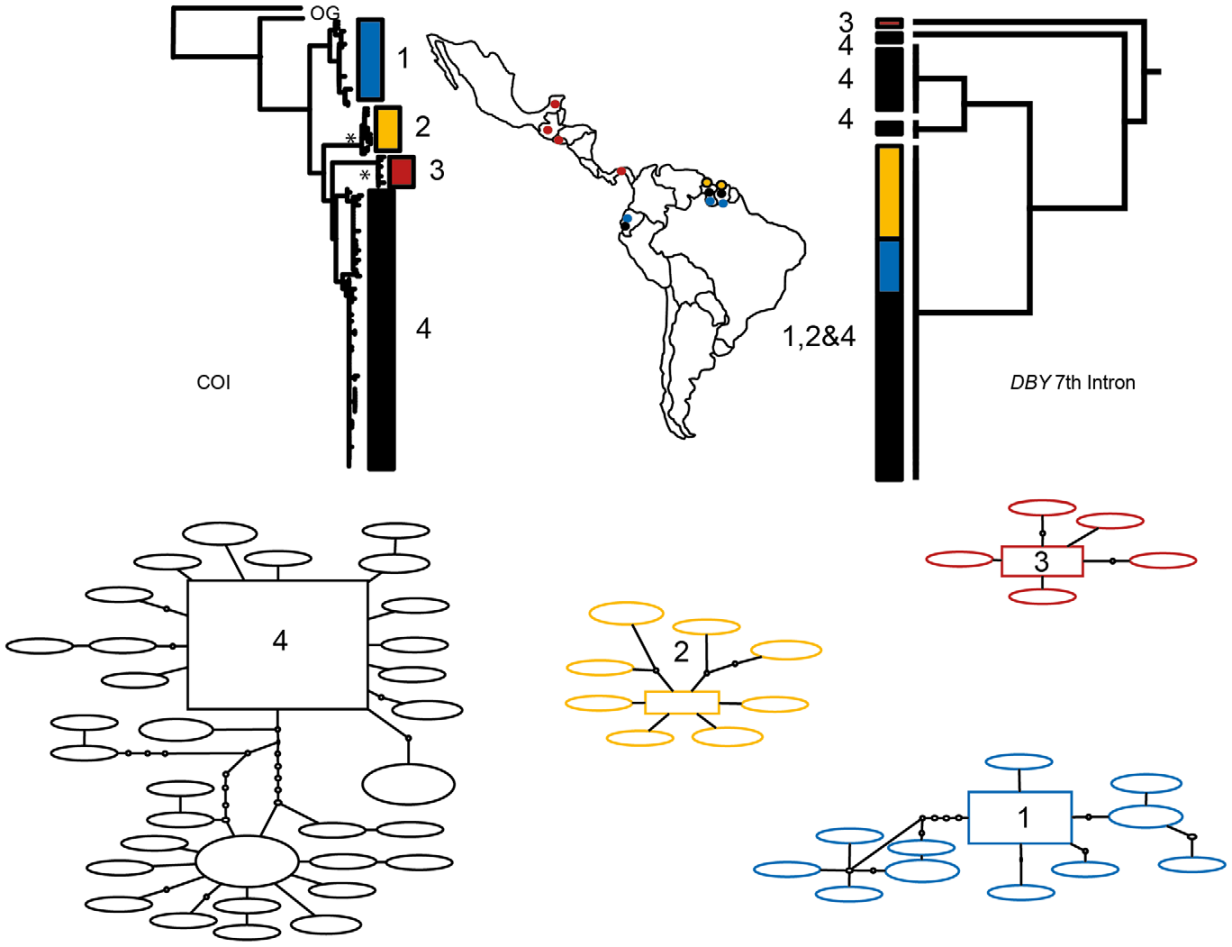
Haplotype maps and phylogenetic reconstruction of COI and the 7^th^ intron of the *Dby* gene in *Platyrrhinus helleri* with sampling distributions. In the statistical parsimony networks each circle represents a single haplotype with circle size scaled by haplotype frequency. Squares indicate the most common haplotype in the network. The COI tree represents the maximum likelihood phylogeny of mitochondrial lineages. Branch supports represent non-parametric Shimodaira-Hasegawa-like (SH-like) values, those equal or greater than 95% are indicated with an asterisk. Y-chromosome intron data is depicted using a UPGMA diagram. Colour coding of branches and haplotype networks matches the sampling distribution.

In *Uroderma bilobatum* (Figure 7), *Micronycteris megalotis* (Figure 8) and *Platyrrhinus helleri* (Figure 9) numerous unconnected intraspecific mitochondrial haplotype networks were indicated. In all cases, the *Dby* 7^th^ intron region was polymorphic however, the observed Y-chromosome groups were not congruent with those identified in COI. As in *G. soricina*, COI haplotype networks for *U. bilobatum* were not congruent with the ML phylogeny. In this case, specimens from Ecuador are largely contained within the South American network and phylogenetic lineage but one has a problematic placement as either connected to the Central American network or forming an outgroup to the rest of the South American specimens in the phylogenetic reconstruction. In addition, specimens from Central America and one from Ecuador form a single haplotype network but are not recovered as reciprocally monophyletic in the phylogeny (with or without the inclusion of the unusually placed sample from Ecuador). In *U. bilobatum* the *Dby* 7^th^ intron polymorphisms were restricted to two unusual haplotypes within the South American group and the remaining 57 sequences were monomorphic.

## Discussion

In this study I test for conflict or congruence in patterns of sequence divergence between the maternally inherited mitochondrial partial COI sequence and the paternally inherited *Dby* 7^th^ intron region of the Y-chromosome. Using these data I differentiate cases where I cannot exclude phylogeographic structuring in cohesive species from cases where gene flow has ceased and cryptic speciation is likely. Both gene regions show variation consistent with current taxonomic designations and reveal additional intraspecific lineages which may represent previously unrecognized species. In three of the eight tested morpho-species, well-supported mitochondrial groups are also defined by unique fixed characters in the *Dby* 7^th^ intron region though ongoing hybridization between two sympatric groups of *G. soricina* is possible. In the other five cases, the *Dby* 7^th^ intron is either invariant or the polymorphisms do not correspond to mitochondrial structure. Congruence in gene flow patterns between COI and the Y-chromosome intron region cannot be predicted from any measure of mitochondrial sequence divergence, either within recognized species or between putative cryptic species, suggesting that, if these regions are indicating species limits, mitochondrial sequence divergence alone is not a useful predictor of gene flow in these taxa.

### Congruence between maternally and paternally inherited genes – a case for speciation

In three species, *Chrotopterus auritus*, *Glossophaga soricina* and *Saccopteryx bilineata*, genetic groups recognized in the maternally inherited COI also showed sequence divergence at the paternally inherited *Dby* 7^th^ intron region. The congruence between these genes strongly supports the existence of cryptic species in these taxa, particularly if evaluated using the genetic species concept [6]. *Glossophaga soricina* requires additional scrutiny as groups 1 and 3 each contains two distinct haplotypes of the *Dby* 7^th^ intron region suggesting either incomplete lineage sorting and insufficient time since divergence for resolution or ongoing hybridization between these two groups.

### Incongruence between maternally and paternally inherited genes

In the remaining cases, groups recognized using COI were not distinguishable at the *Dby* 7^th^ intron region. In *D. rotundus* and *T. cirrhosus* the intron was monomorphic, thus it does not offer support for the status of the mitochondrial groups in these species though it also does not suggest an alternative topology. Greater genetic variation in COI than the *Dby* 7^th^ intron sequences is not surprising if the latter region is evolving at a slower rate than COI. In addition, branch support values for the phylogenetic reconstruction of *D. rotundus* lineages are weak and the topology of the ML and Bayesian reconstructions are inconsistent. Mitochondrial DNA is highly polymorphic in *Desmodus* [15], [40], [41] and may not carry enough phylogenetic signal to recover the arrangement of these groups (the recovery of group 1 in the ML phylogeny was problematic). Previous hypotheses [41] suggest at least five distinct groups matching closely to the haplotype networks and phylogenetic reconstructions presented here (Figure 5), though the placement of samples from the São Paulo region of Brazil is inconsistent in both these and other published reconstructions. In addition, the phylogeny presented by Martins et al. [41] shows substructure in the identified groups, particularly in samples from the Amazon and Cerrado, which may match the distinct networks recovered from samples from the Guyana Shield in this study. However, phylogeographic patterns in *Desmodus* may be complicated by their preference for domesticated livestock [42], conferring a tendency to follow human settlements and resulting in recent rapid population expansion and concomitant mixing of previously isolated populations [15].

The *Dby* 7^th^ intron region of *Uroderma bilobatum*, *Micronycteris megalotis* and *Platyrrhinus helleri* is polymorphic, but does not match the structure in COI. *M. megalotis* is genetically diverse though counting the exact number of lineages varies with methodology [12] and may be limited by small sample size. If the mitochondrial structure observed here does not represent cryptic taxa, but population level phenomena with continual gene flow, the molecular diversity in this species is large (mean = 4.2%, range 0–7.7% K2P) and among the highest measured in bat species where cryptic speciation is not likely [12]. There was some evidence of fixed substitutions between groups but specimens of *M. megalotis* are relatively rare in the museum collection and a very small sample size prevents a more thorough analysis. Additionally, in *Micronycteris megalotis* and *Platyrrhinus helleri* intraspecific groups appear to occupy largely sympatric ranges further suggesting speciation has or is occurring. It is very possible that slower divergence of the Y-chromosome region has led to incomplete lineage sorting in these taxa but further evidence is required.

*Uroderma bilobatum* is a particularly problematic taxon. Three mitochondrial lineages have been previously identified which are supported by chromosomal changes leading to distinct karyotypes [8] (two of these groups are included in this survey). My phylogenetic analysis fails to recover these though the reconstruction has poor branch support in general and the arrangements appear to be sensitive to the choice of outgroup. My haplotype networks more closely correspond to these previously recognized groups as samples from Ecuador are connected with the South American network (with the exception of one specimen). The three previously identified lineages have likely formed from geographic isolation and the fixation of genetic features [8] and introgression between mitochondrial lineages was observed to be minimal [8]. The two groups in common between Hoffman et al. [8] and this analysis lack distinguishing features at the *Dby* 7^th^ intron region. In *U. bilobatum*, most Y-chromosome sequences were identical while two sequences contained numerous mutations. These two sequences may represent the retention of ancestral haplotypes, novel mutations or even a rare amplification of the X-chromosome homologue to the *Dby* gene (see below). Excluding these two unusual sequences, the remaining 57 Y-chromosome sequences from *U. bilobatum* follow the monomorphic pattern of *T. cirrhosus* and *D. rotundus*. More data are required to understand the diversity of the *Dby* 7^th^ intron region in this species but if *U. bilobatum* is a species complex [8] this suggests that lack of divergence at the *Dby 7^th^* intron region cannot be used as evidence against cryptic speciation. Given this, speciation in *T. cirrhosus* and *D. rotundus* remains a distinct possibility and additional lines of evidence are required.

### Species Diagnosis

Numerous methods of genetic species delimitation have been proposed [43] which rely on either “distance” or “character” approaches [44]. Distance-based approaches can suffer from substitution saturation, leading to under-diagnosis of sequence differences, and lend themselves to similarity cut-offs [44] or thresholds [45], [46], which are unlikely to be applicable outside a narrow range of taxa [47]. Character-based analyses are similar to traditional taxonomic approaches but can be hindered by a lack of analytical platforms to aid character detection, and suffer when fast mutation rates obscure synapomorphies and generate homoplasies. Character-based approaches also rely on the supposition that additional sampling will not reveal new polymorphisms at diagnostic sites, an assumption that cannot be empirically evaluated.

While COI, with no indels known for most vertebrates, can be analyzed either way, the highly polymorphic nature of COI in bats [12] makes a character-based approach impractical. In contrast, the *Dby* 7^th^ intron region is less polymorphic but contains many indels, often of substantial size, making alignments for distance-based analyses and tree construction difficult. For that reason, in this study I employ a distance-based approach for the COI data and a character-based approach for the interpretation of *Dby* 7^th^ intron data (Figure 4). My sample sizes for Y-chromosome data in each species are variable (n = 20–64), and those with consistent COI and Y-chromosome patterns were large (*C. auritus* n = 33, *G. soricina* n = 62, *S. bilineata* n = 39) though some specific groups are not well sampled (some were represented by females only and are thus not evaluated) and additional taxonomic scrutiny is required particularly in contact zones where hybridization is possible. While alignments are useful for analysis, they are difficult to display visually thus I show only relevant sections from a representative sample of individuals in Figure 4 and use UPGMA trees in all other figures. The *Dby* 7^th^ intron region used here is easy to amplify across a wide variety of taxa [28] but caution is required in interpreting the *Dby* region as, while fast, it does not appear to evolve as fast as mtDNA. Like mitochondrial genes, Y-chromosomal DNA outside the pseudo-autosomal region may also be subject to selective sweeps reducing variability. In addition, a homologous region to the *Dby* gene has been identified on the X-chromosome in some species though it is substantially divergent [48]. While I observed no evidence of co-amplification from the male X-chromosome, and these primers may preferentially bind to the target area, to the best of my knowledge no rigorous analysis of the specific amplification and variability of these homologous regions has been conducted in bats.

While ML and Bayesian trees were largely congruent, subtle differences in the arrangements were apparent (Figure S1). The goal of this analysis was not phylogenetic reconstruction but taxonomic assessment (thus it is the number of branches on the trees not their arrangement which is important) and phylogenies presented here should be considered tentative. Additional gene segments will be required to establish robust phylogenetic hypotheses if species complexes receive additional support in future analyses.

### Geographic Considerations

Distinct mitochondrial lineages are found with both allopatric and sympatric contemporary ranges with respect to other intraspecific lineages. While the power of the GSC is that it can be applied to allopatric populations, the existence of sympatric lineages in *G. soricina* with evidence of hybridization suggests reproductive isolation is not complete and that this complex may not meet the conditions of the biological species concept [25]. In the cases of *C. auritus* and *S. bilineata*, hybridization was not observed but in both cases at least one group had a small sample size and the distributions were non-overlapping leaving the genetic status of these complexes at contact zones unclear. If groups at contact zones maintain the same pattern observed here they may meet the criteria for both the genetic [6] and biological [25] species concepts. Alternatively, limited introgression in hybrid zones would suggest speciation is ongoing and will provide a unique opportunity to investigate the mechanisms of speciation and reinforcement.

The most consistently observed genetic split is between a Central American group and a South American group. This geographic structure is common in land vertebrates and has both phylogeographic and taxonomic implications. The formation of the Andean mountain range over successive geological uplifts [49] has resulted in numerous vicariant events resulting in reduced or complete cessation of gene flow in separated populations. Patterns of morphological and genetic differentiation in this region have led to the description of a number of new bat species including *Carollia sowelli* from the South American sister species *C. brevicauda sensu stricto* [7] and a number of subspecies e.g. *U. bilobatum davisi* [50] *G. soricina handleyi* [51] and *C. auritus auritus* [52] from Central America which are distinguished by morphological characters but may match the genetic groups identified here.

The inclusion of samples from Ecuador in both Central and South American groups demonstrates the highly complex geographic history of this area and reflects the great faunal interchange followed by the rise of the Central American land bridge approximately 3.5 million years BCE [29]. Though many volant species would have been gradually dispersing before the formation of the bridge was complete, little is known about the dispersal patterns of bats during this period [29]. The rise of the northern Andes occurred in several waves, including the uplift of the Western and Central Cordillera in the late Cretaceous-Paleocene and the Eastern Cordillera during the Pliocene-and early Holocene [49], trapping groups on either side of this barrier from ancient through to modern times. As a result, the western portion of Ecuador may receive migrants from Central America before any other area of South America, but may also contain recent dispersers from South American groups or a combination of scenarios influenced by species' vagility and habitat preferences. Robust, multi-gene phylogenies will be required to elucidate the specific biogeographical scenarios for each of the groups considered here.

### Speciation and the genetic species concept

The genetic species concept for mammals as described by Baker and Bradley [6] requires that species status be extended to any group which maintains the integrity of its gene pool, a pattern indicated for a number of the groups here. Baker and Bradley [6] do not establish operational criteria to identify putative species though Bradley and Baker [9] provide guidelines for typical levels of mean cytochrome *b* sequence divergences at different taxonomic levels. While intraspecific divergence for seven of the eight species investigated here falls into the range of likely speciation [9] only three cases were supported by both male and female gene flow patterns. In addition, the only case that has been identified as a species complex previously [8], *U. bilobatum*, had the lowest mean intraspecific divergence measured here and would not have been highlighted using the Bradly and Baker [9] criteria. Mean values are difficult to interpret and give no indication of the variability, maximum diversity or divergence between putative groups. While groups that were not well supported were on average less genetically divergent there was substantial overlap with supported groups negating the use of any specific cut-off for species recognition (Table 2).

I used markers with the most disparate modes of inheritance possible (maternal vs. paternal non-recombining regions) to test species boundaries. These two markers are unlikely to show convergence by chance. Though I did not observe congruence in all cases, these data do not rule out speciation among mitochondrial groups recognized in *D. rotundus*, *T. cirrhosus*, *U. bilobatum*, *M. megalotis* and *P. helleri* and, in fact, speciation is still a reasonable hypothesis particularly if the Y-chromosome region is evolving more slowly than COI and sufficient time has not passed for divergence at the Y-chromosome locus. If future research provides evidence suggesting speciation has not occurred it would indicate that these species have extraordinarily high levels of mitochondrial diversity. It seems likely that unrecognized species exist in *C. auritus*, *G. soricina* and *S. bilineata*. Additional taxonomic scrutiny will be required to identify additional characters which can separate these putative species in the field, however the findings here are inline with predictions that many Neotropical bat species remain undescribed [6], [10]–[12] even among frequently encountered taxa.

### Conclusions

This study examines whether mitochondrial genetic patterns suggesting cryptic speciation are congruent with patterns in the paternally inherited *Dby* 7^th^ intron region of the Y-chromosome. Independently evolving lineages supported by both genes exist in *C. auritus*, *G. soricina* and *S. bilineata* suggesting that reproductive isolation may also exists between lineages. The *Dby* 7^th^ intron did not show variation consistent with the proposed species complexes in *D. rotundus*, *T. cirrhosus*, *U. bilobatum*, *P. helleri* or *M. megalotis*. While I cannot exclude ongoing gene flow between intraspecific lineages in these species, divergences are very high and many intraspecific groups appear to occupy sympatric distributions strongly suggesting speciation has or is occurring. This investigation supports a number of hypotheses concerning the role of specific geographic barriers, in particular the potential for numerous separate sister taxa pairs in Central and South America. While COI is an excellent diagnostic for confirming the identification of bat species, single locus genetic data are insufficient to resolve the status of hypothesized cryptic species. The careful selection of complementary loci with independent evolutionary histories can provide resolution and a framework for applying the genetic species concept in mammals.

## Supporting Information

Figure S1 A comparison of maximum likelihood and Bayesian phylogenetic reconstructions of the mitochondrial COI 5′ region.
(PDF)

Table S1 GenBank and BOLD accessions for all COI and *Dby* 7^th^ intron sequences. Museum accessions for all vouchered specimens.
(XLS)

## References

[1] Willig MR, Selcer KW (1989). Bat species density gradients in the new world: a statistical assessment.. J Biogeogr.

[2] Simmons NB, Voss RS (1998). The mammals of Paracou, French Guiana, a Neotropical lowland rainforest fauna Part 1.. Bats B Am Mus Nat Hist.

[3] Barquez RM, Diaz MM (2001). Bats of the Argentine Yungas: a systematic and distributional analysis.. Acta Zool Mex.

[4] Lim BK, Engstrom MD (2001). Species diversity of bats (Mammalia: Chiroptera) in Iwokrama Forest, Guyana and the Guianan subregion: implications for conservation.. Biodivers Conserv.

[5] Lim BK, Wagner AP, Passos FC (2003). Differentiation and species status of the Neotropical yellow eared bats *Vampyressa pusilla* and *V. thyone* (Phyllostomidae) with a molecular phylogeny and review of the genus.. Acta Chiropterol.

[6] Baker RJ, Bradley RD (2006). Speciation in mammals and the Genetic Species Concept.. J Mammal.

[7] Baker RJ, Solari S, Hoffmann FG (2002). A new Central American species from the *Carollia brevicauda* complex.. Occas Pap The Museum of Texas Tech University.

[8] Hoffmann FG, Owen JG, Baker RJ (2003). mtDNA perspective of chromosomal diversification and hybridization in Peters' tent-making bat (*Uroderma bilobatum*: Phyllostomidae).. Mol Ecol.

[9] Bradley RD, Baker RJ (2001). A test of the genetic species concept: cytochrome-*b* sequences and mammals.. J Mammal.

[10] Clare EL, Lim BK, Engstrom MD, Eger JL, Hebert PDN (2007). DNA barcoding of Neotropical bats: species identification and discovery within Guyana.. Mol Ecol Notes.

[11] Borisenko AV, Lim BK, Ivanova NV, Hanner RH, Hebert PDN (2008). DNA barcoding in surveys of small mammal communities: a field study in Suriname.. Mol Ecol Resources.

[12] Clare EL, Lim BK, Fenton MB, Hebert PDN (2011). Neotropical bats: Estimating species diversity with DNA barcodes.. PLoS one.

[13] Hebert PDN, Cywinska A, Ball SL, deWaard JR (2003). Biological identifications through DNA barcodes.. P Roy Soc B-Biol Sci.

[14] Haffer J (1969). Speciation in Amazonian forest birds.. Science.

[15] Ditchfield AD (2000). The comparative phylogeography of Neotropical mammals: patterns of intraspecific mitochondrial DNA variation among bats contrasts to nonvolant small mammals.. Mol Ecol.

[16] Bohonak AJ (1999). Dispersal, gene flow, and population structure.. Q Rev Biol.

[17] Hoffmann FG, Baker RJ (2003). Comparative phylogeography of short-tailed bats (*Carollia*: Phyllostomidae).. Mol Ecol.

[18] Koopman KF, Mares MA, Genoways HH (1981). Biogeography of the bats of South America.. University of Pittsburgh.

[19] Cracraft J, Prum RO (1988). Patterns and processes of diversification: speciation and historical congruence in some Neotropical birds.. Evolution.

[20] Mayr E (1942). Systematics and the origin of species.

[21] Berthier P, Excoffier L, Reudi M (2006). Recurrent replacement of mtDNA and cryptic hybridization between two sibling bat species *Myotis myotis* and *Myotis blythii*.. P Roy Soc B-Biol Sci.

[22] Webb NJ, Tidemann CR (1995). Hybridization between black (*Pteropus alecto*) and grey-headed (*P. poliocephalus*) flying-foxes (Megachiroptera: Pteropodidae).. Australian Mammalogy.

[23] Mao X, Zhang J, Zhang S, Rossiter SJ (2010). Historical male-mediated introgression in horseshoe bats revealed by multilocus DNA sequence data.. Mol Ecol.

[24] Gavrilets S (2004). Fitness landscapes and the origin of species.

[25] Coyne JA, Orr HA (2004). Speciation.

[26] Hebert PDN, Penton EH, Burns JM, Janzen DH, Hallwachs W (2004). Ten species in one: DNA barcoding reveals cryptic species in the neotropical skipper butterfly *Astraptes fulgerator*.. P Natl Acad Sci USA.

[27] Smith MA, Woodley NE, Janzen DH, Hallwachs W, Hebert PDN (2006). DNA barcodes reveal cryptic host-specificity within the presumed polyphagous members of a genus of parasitoid flies (Diptera: Tachinidae).. P Natl Acad Sci USA.

[28] Lim BK, Engstrom MD, Bickham JW, Patton JC (2008). Molecular phylogeny of New World sheath-tailed bats (Emballonuridae: Diclidurini) based on loci from the four genetic transmission systems of mammals.. Biol J Linn Soc.

[29] Lim BK (2007). Divergence times and origin of Neotropical sheath-tailed bats (Tribe Diclidurini) in South America.. Mol Phylogenet Evol.

[30] Kimura M (1980). A simple method for estimating evolutionary rates of base substitutions through comparative studies of nucleotide sequences.. J Mol Evol.

[31] Tamura K, Dudley J, Nei M, Kumar S (2007). MEGA4: Molecular Evolutionary Genetics Analysis (MEGA) software version 4.0.. Mol Biol Evol.

[32] Clement M, Posada D, Crandall KA (2000). TCS: a computer program to estimate gene genealogies.. Mol Ecol.

[33] Posada D, Crandall KA (1998). MODELTEST: testing the model of DNA substitution.. Bioinformatics.

[34] Swofford DL (2000). PAUP*..

[35] Guindon S, Gascuel O (2003). A simple, fast, and accurate algorithm to estimate large phylogenies by maximum likelihood.. Syst Biol.

[36] Huelsenbeck JP, Ronquist F (2001). MrBayes: Bayesian inference of phylogenetic trees.. Bioinformatics.

[37] Kumar S, Tamura K, Nei M (2004). MEGA3: integrated software for molecular evolutionary genetics analysis and sequence alignment.. Brief Bioinform.

[38] Hasegawa M, Kishino H, Yano T-A (1985). Dating of the human-ape splitting by a molecular clock of mitochondrial DNA.. J Mol Evol.

[39] Tamura K, Nei M (1993). Estimation of the number of nucleotide substitutions in the control region of mitochondrial DNA in humans and chimpanzees.. Mol Biol Evol.

[40] Martins FM, Ditchfield AD, Meyer D, Morgante JS (2007). Mitochondrial DNA phylogeography reveals marked population structure in the common vampire bat, *Desmodus rotundus* (Phyllostomidae).. J Zool Syst Evol Res.

[41] Martins FM, Templeton AR, Pavan ACO, Kohlback BC, Morgante JS (2009). Phylogeography of the common vampire bat (*Desmodus rotundus*): Marked population structure, Neotropical Pleistocene vicariance and incongruence between nuclear and mtDNA markers.. BMC Evol Biol.

[42] Voigt CC, Kelm DH (2006). Host preferences of the common vampire bat (*Desmodus rotundus*; Chiroptera) assessed by stable isotopes.. J Mammal.

[43] Sites JW, Marshall JC (2003). Delimiting species: a renaissance issue in systematic biology.. Trends Ecol Evol.

[44] DeSalle R, Egan MG, Siddall M (2005). The unholy trinity: taxonomy, species delimitation and DNA barcoding.. Philos T R Soc B.

[45] Hebert PDN, Stoeckle MY, Zemlak TS, Francis CM (2004). Identification of birds through DNA barcodes.. PLOS Biol.

[46] Kerr KCR, Stoeckle MY, Dove CJ, Weigt LA, Francis CM, Hebert PDN (2007). Comprehensive DNA barcode coverage of North American birds.. Mol Ecol Notes.

[47] Cognato AI (2006). Standard percent DNA sequence differences for insects does not predict species boundaries.. J Econ Entomol.

[48] Krausz C, McElreavey K (1999). Y Chromosome and male infertility.. Front Biosci.

[49] Gregory-Wodzicki KM (2000). Uplift history of the Central and Northern Andes: A review.. Geol Soc Am Bull.

[50] Baker RJ, McDaniel VR (1972). A new subspecies of *Uroderma bilobatum* (Chiroptera: Phyllostomatidae) from Middle America.. Occ Pap The Museum of Texas Tech University.

[51] Alvarez J, Willig MR, Jones JK, Webster WD (1991). *Glossophaga soricina*.. Mammalian Species.

[52] Medellin RA (1989). *Chrotopterus auritus*.. Mammalian Species.

