# Supplementary material for: Cryptic Species? Patterns of Maternal and Paternal Gene Flow in Eight Neotropical Bats
Source: PLoS One. 2011 Jul 26;6(7):e21460. doi: 10.1371/journal.pone.0021460 (PMC3144194; doi:10.1371/journal.pone.0021460)
Supplement: Figure S1 — A comparison of maximum likelihood and Bayesian phylogenetic reconstructions of the mitochondrial COI 5′ region. (PDF) [file pone.0021460.s001.pdf]

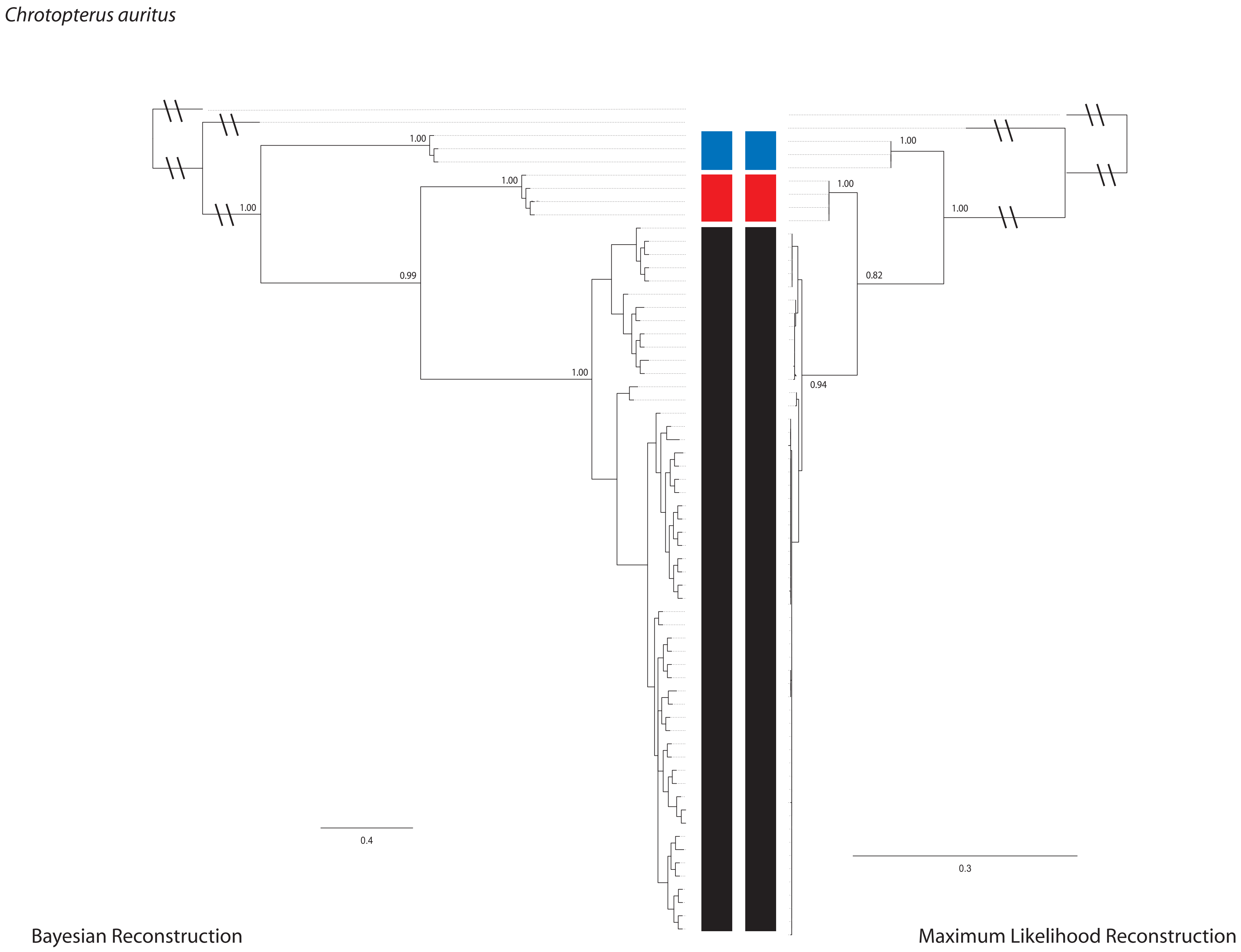
Chrotopterus auritus
1.00
1.00
1.00
0.99
1.00
0.4
Bayesian Reconstruction
1.00
1.00
1.00
0.82
0.94
0.3
Maximum Likelihood Reconstruction

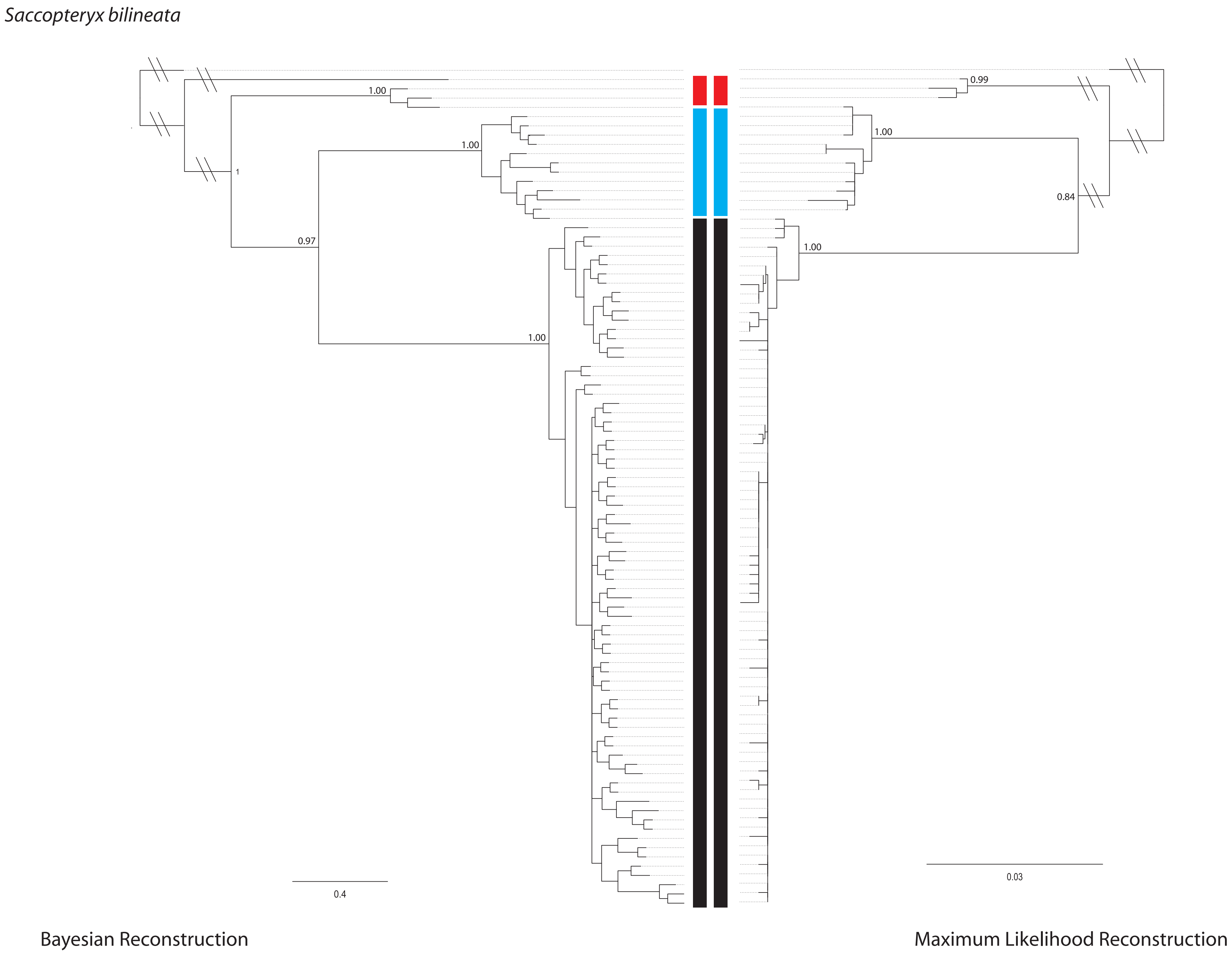

Saccopteryx bilineata
1.00
1.00
1
0.97
1.00
0.4
0.99
1.00
0.84
1.00
0.03
Bayesian Reconstruction
Maximum Likelihood Reconstruction

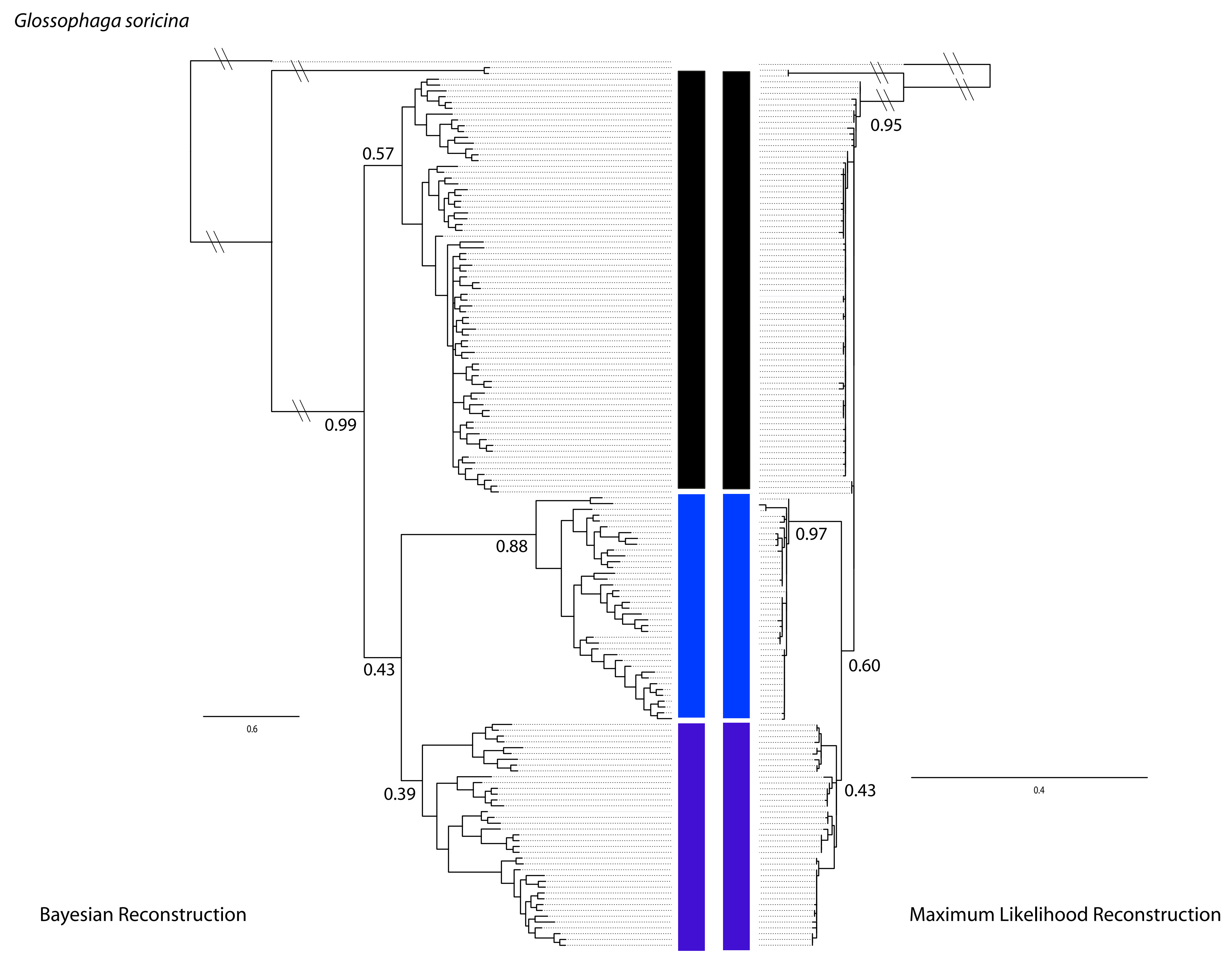
Glossophaga soricina
0.57
0.99
0.88
0.43
0.39
0.6
Bayesian Reconstruction
0.95
0.97
0.60
0.43
0.4
Maximum Likelihood Reconstruction

Desmodus rotundus

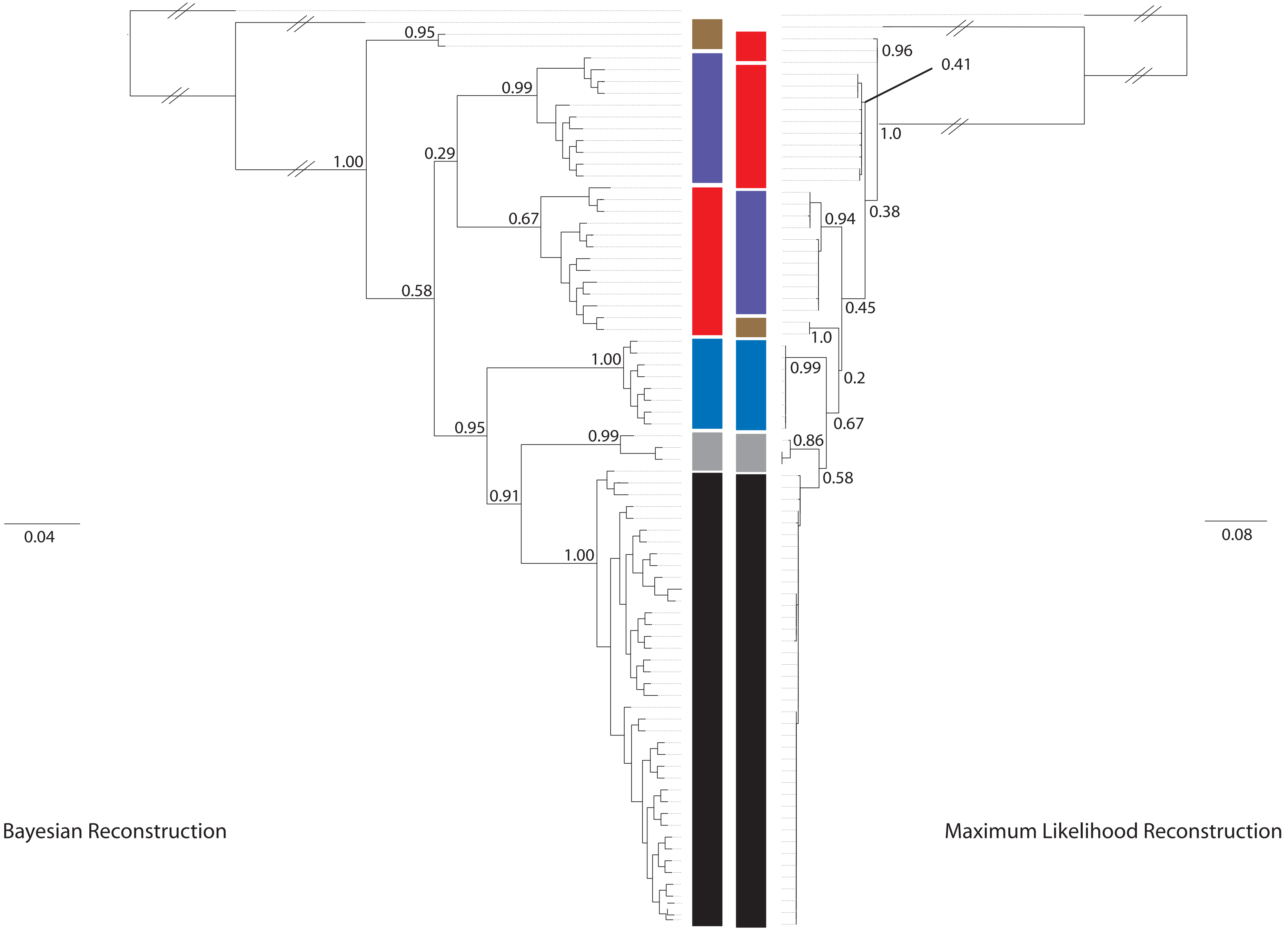

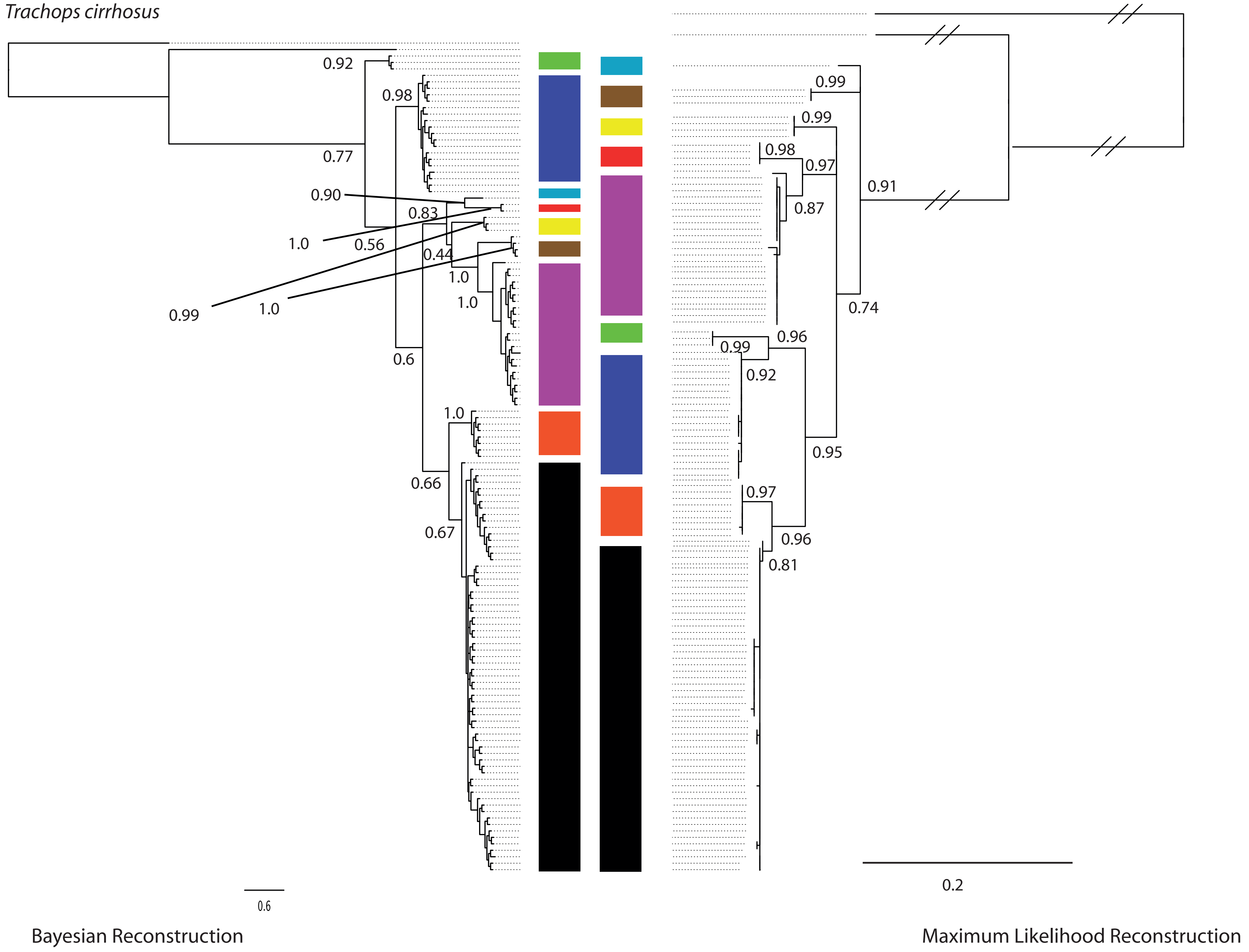
Trachops cirrhosus
0.92
0.98
0.77
0.90
0.83
1.0
0.56
0.44
1.0
0.99
1.0
1.0
0.6
1.0
0.66
0.67
0.6
Bayesian Reconstruction
0.99
0.99
0.98
0.97
0.91
0.87
0.74
0.96
0.99
0.92
0.95
0.97
0.96
0.81
0.2
Maximum Likelihood Reconstruction

Uroderma bilobatum

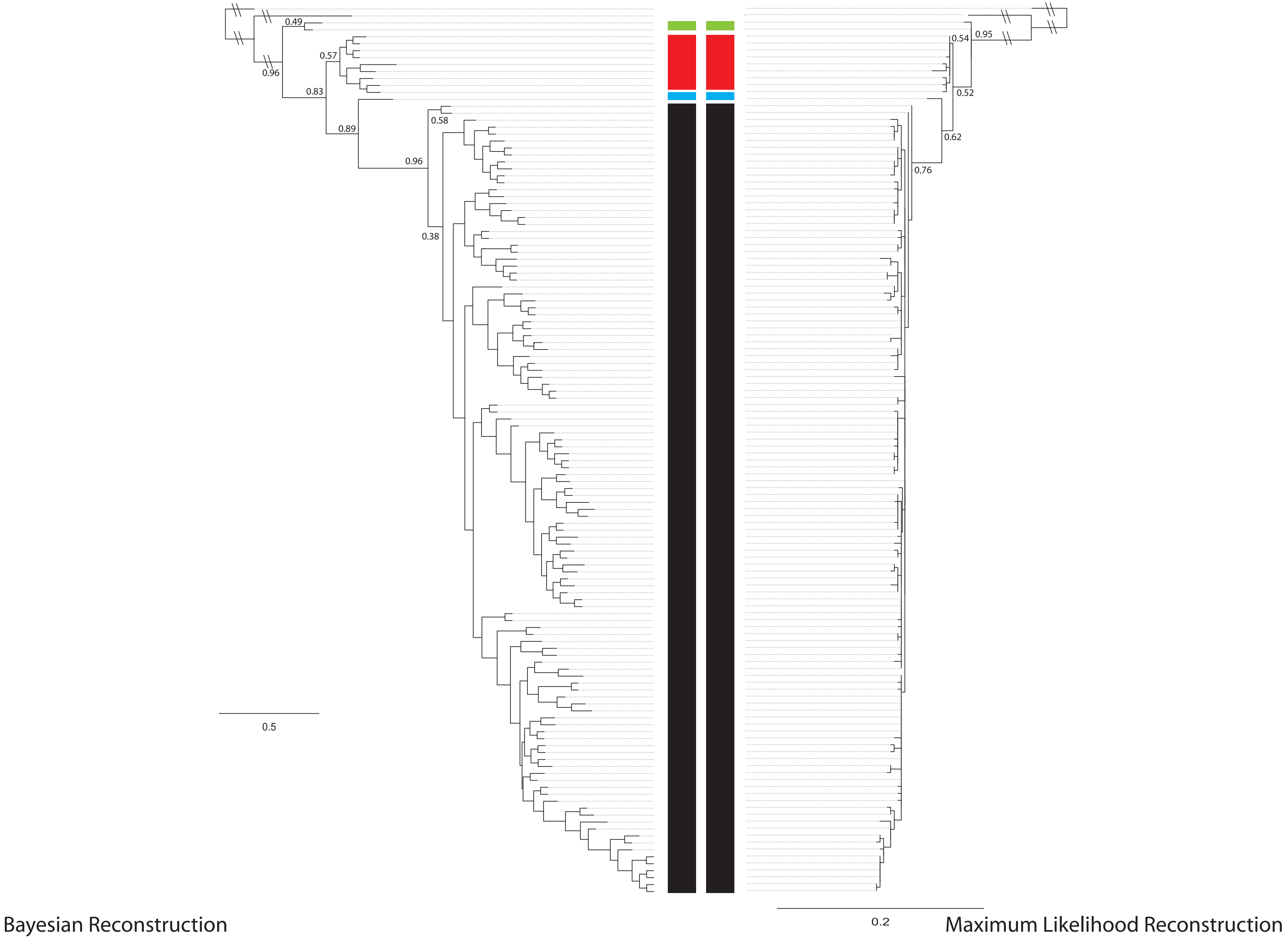

Micronycteris megalotis

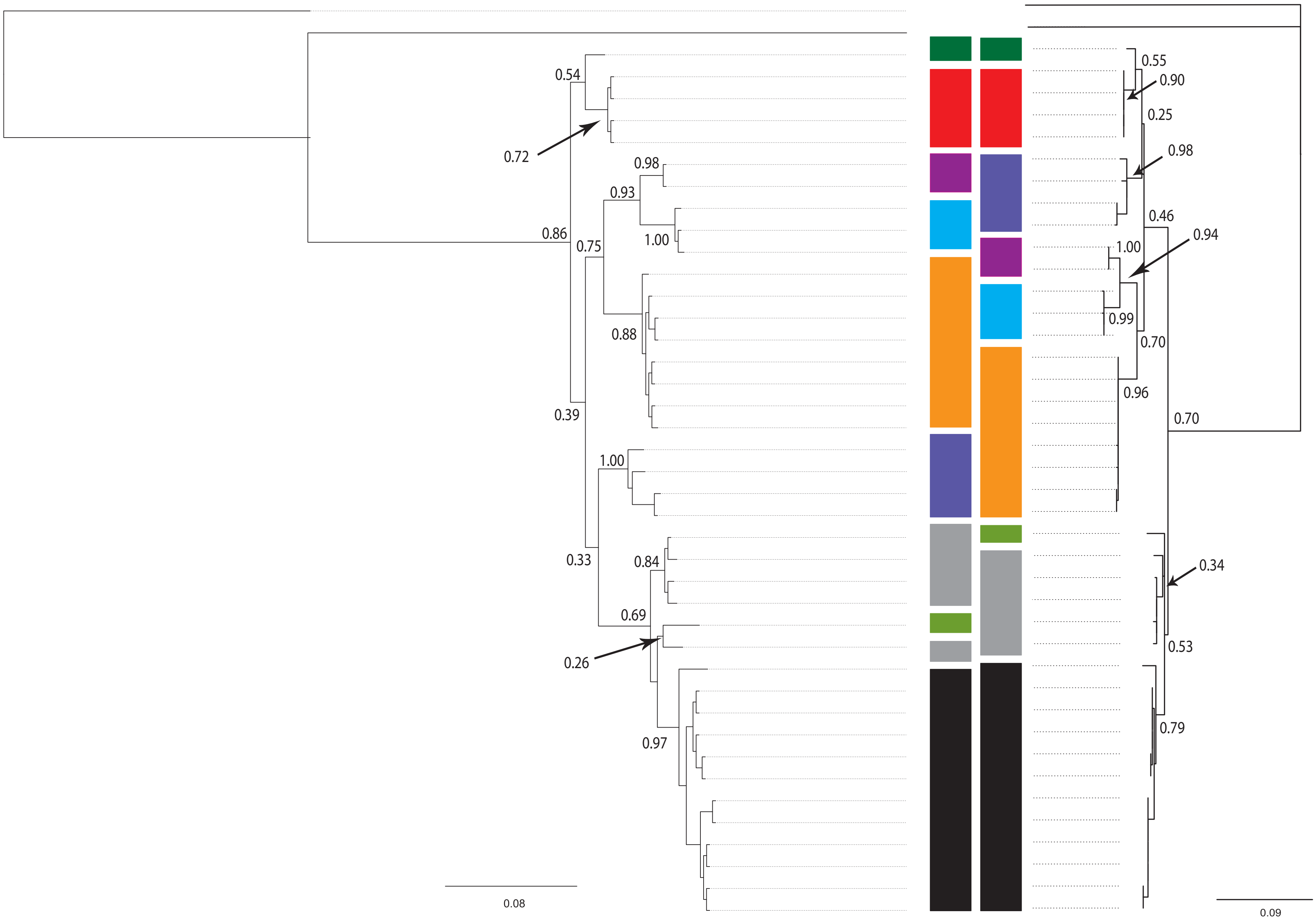

Bayesian Reconstruction

Maximum Likelihood Reconstruction

Platyrrhinus helleri

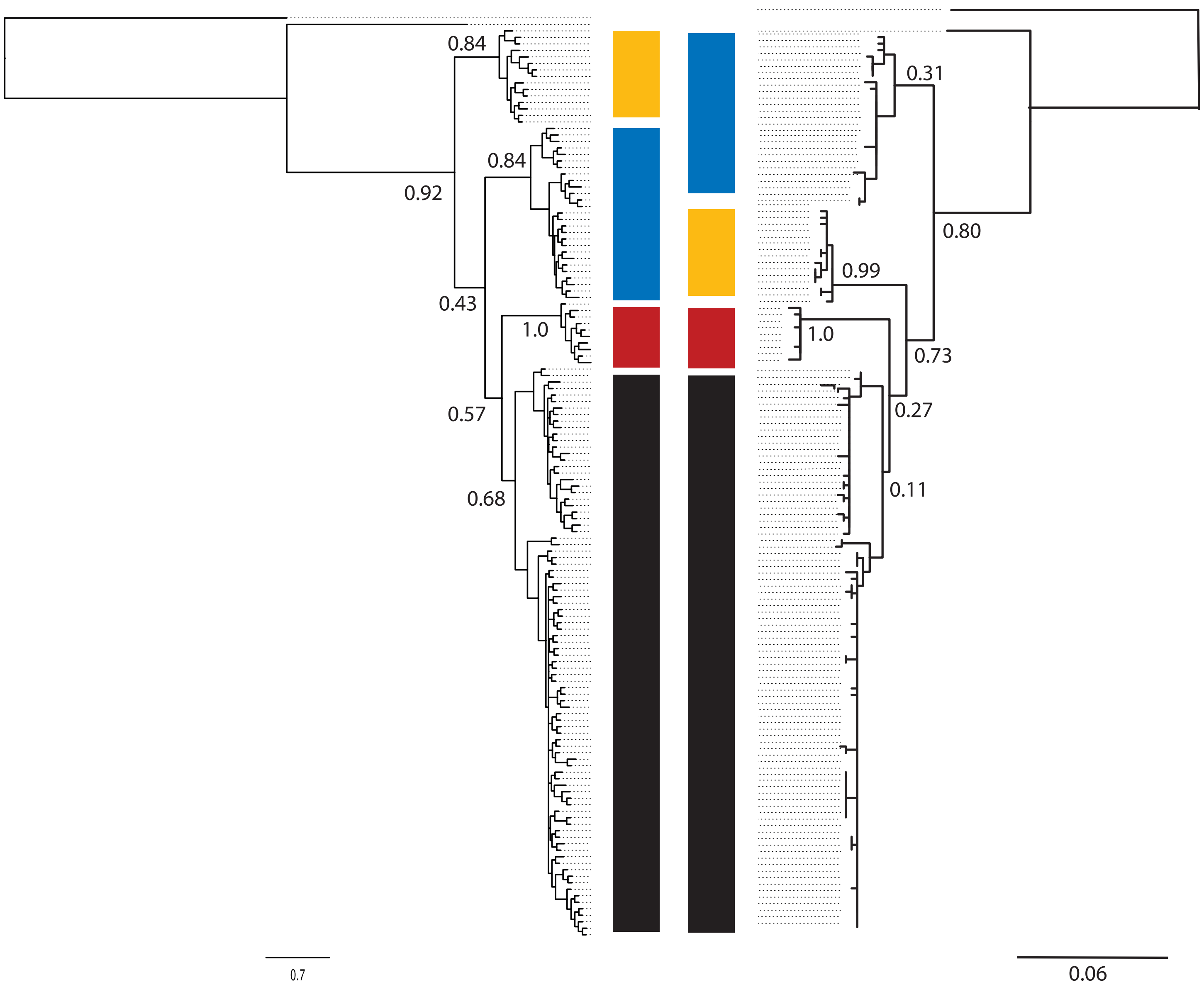

Bayesian Reconstruction

Maximum Likelihood Reconstruction
